# Report of the Diseases of Edinburgh for December, 1809

**Published:** 1810-02

**Authors:** John Roberton


### *Report of the Diseases of Edinburgh for December,* 1809.

By JOHN ROBERTON, M. D.

For several days after the commencement of the month the weather was variable, frost and thaw alternating with each other frequently in the course of one day. About the end of the last third of the month, however, the weather became more regularly of a tempestuous nature, and we were, for several days, almost constantly assailed either by hard gales of very cold wind, by falls of snow, or by torrents of rain; at other times all these prevailed at once, forming a most tremendous hurricane.

During the latter half of the month the weather was less tempestuous, but very inconstant. Frost, rain, and slight falls of snow, continued to alternate with each other, till its termination.

The barometer was in general low, especially in the earlier part of the month. During some of the more favourable days, however, nearer the end of it, the barometer, for a few hours only, rose considerably.

The thermometer stood in general about 40, or from that to 45 degrees. The few frosty days, however, which we occasionally had near the end of the month, reduced it much lower.

The most prevalent diseases of the month have been of an inflammatory nature, fevers, croup, and small-pox.

It is almost an universal rule for the greatest inflammatory state of the general system to commence with the cold weather, and terminate in the course of the spring months. Thus various inflammatory diseases exist in various individuals, the same cause producing these different effects; partly from the immediate particular habits in eating, drinking, &c. So far, however, as the winter season has advanced, vanced, the usual inflammatory disposition has not been nearly so conspicuously marked as in almost every previous year. To account for this, is perhaps impossible. The weather, to the particular nature of which we have been in the habit of, in a great measure, ascribing these complaints, has not been, in any remarkable degree, different from what is usual at the same period of the season. From former reports also, it will be observed, that last year, after the inflammatory complaints began as usually to abate, they again, even when the warm weather had commenced, became much more general, and in many instances more fatal than even during their prevalence in the previous winter months. It would therefore appear that there are some other causes besides what have been enumerated, tending to increase the inflammatory disposition.

Although, as observed, these inflammatory complaints have not been nearly so general as usual, yet they have not been by any means entirely wanting. In practice we meet with some very severe cases of pneumonia, enteritis, and the inflammatory or first stage of hydrocephalus internus. Catarrh and acute rheumatism are also occasionally met with.

I have seen a few cases of pneumonia of uncommon severity, which were cured only where the most active means were employed. In two of these I found it necessary every day, for ten days, to order large bleedings; blisters over the whole forepart of the thorax, and saline cathartics, to be repeatedly administered. One of these patients recovered completely, the other seemingly recovered, but in a few days effusion of fluid into the thorax and abdomen took place, which, notwithstanding every means that could be used, terminated fatally. The generality of the few cases of this complaint which have appeared here, yielded, with great facility, to the common practice.

Enteritis has, in almost every instance, yielded almost immediately after the effects of a blister applied over the abdomen had been produced.

The first or inflammatory stage of hydrocephalus internus has been more common than either of the two formerly mentioned complaints, but early attention to the state of the pulse and the bowels almost uniformly prevent effusion into the ventricles of the brain. The application of the cap blister, the administration of purgatives till the stools (which as an occasional accompanying symptom of this disease are in general green or black) become become of a natural colour; and the application of leeches to the temples, or even, in severe cases, opening the jugular vein or temporal artery, are followed by the happiest effects.

Catarrh, which indeed is scarcely ever absent from this city, has been very common; but, like other inflammatory affections, not very severe. Purgative medicines, bathing the feet and legs in warm water before going to bed, and the occasional use of diluting and mucilaginous drinks, have always removed it. Acute rheumatism has also appeared in various parts of the city, and has been removed by attention to the bowels, by sudorific medicines, and, when the first inflammatory state of the complaint had yielded, by the liberal use of friction with the flesh brush or a coarse cloth. In this, as well as in many other complaints, the applications for its removal are too often, in regard to its stages, indiscriminately applied. Frictions, with heating balsamic substances, are often used from the very first appearance of the symptoms; this, I have no hesitation in asserting, protracts the disease, and not unfrequently contributes to assist the complaint in terminating in chronic rheumatism. Were our dependence, in its early stages, to be placed in cooling laxatives and sudorifics, and when these had relaxed, the application of friction, we should prevent such a termination, and even better effect our purpose in its early stages.

Fevers of the typhoid kind are never absent from this city; and as foulness is the cause of them, the reverse tends to prevent or in a great measure to cure them.

Croup has appeared among children; some cases have been very severe, and have even proved fatal. Like every other disease, this is easily removed when early attended to. Large doses of calomel, with the occasional application of blisters round the throat or over the chest, almost uniformly remove the affection.

The small-pox, disgraceful to state, seems to be gaining ground among us. That mulish stubbornness, which, as in other countries, prevails in no scarcity about this place, is certainly the cause of this. Our countryman, Doctor Smollet, termed this city the hot-bed of genius, but he omitted to state, perhaps from partiality to his own country, that it certainly is a soil much calculated to the production of that obstinacy which is strongly marked among the Scots. This is too often mistaken by my countrymen for independence of thought, while independence of thought thought itself is but rarely felt here. Upon the introduction of any remarkable improvement, such as the cow-pox, or any circumstance where open and liberal sentiment is expected to be expressed, each individual, terrified at hazarding his own opinion, seemingly wishes his neighbour to advance what he has to say; and then, as the majority takes one or the other side of a question, the rest soon espouse the same side. It is this which has retarded many useful improvements, and among others, the universal inoculation of the cow-pox.

*Princes Street.*

